# Supplementary material for: SAW Humidity Sensing with rr-P3HT Polymer Films
Source: Sensors (Basel). 2024 Jun 5;24(11):3651. doi: 10.3390/s24113651 (PMC11175178; doi:10.3390/s24113651)
Supplement: Supplementary file 1 [file sensors-24-03651-s001.zip › sensors-2991616-supplementary.pdf]

Supplement material

SAW humidity sensing with rr-P3HT polymer films

Wiesław Jakubik[1*], Jarosław Wrotniak[2], Cinzia Caliendo[3], Massimiliano Benetti[4], Domenico Cannata[4], Andrea Notargiacomo[3], Agnieszka Stolarczyk [5] and Anna Kaźmierczak-Bałata[1]

[1] Institute of Physics CSE, Silesian University of Technology, 44-100 Gliwice, Poland

[2] Institute of Electronics, Silesian University of Technology, 44-100 Gliwice, Poland

[3] Institute for Photonics and Nanotechnologies, IFN-CNR, 00133 Rome, Italy

[4]Institute of Microelectronics and Microsystems, CNR, 00133 Rome, Italy

[5] Department of Physical Chemistry and Technology of Polymers, Silesian University of Technology, 44-100 Gliwice, Poland

*Correspondence: wieslaw.jakubik@polsl.pl; Tel.: +48 322372024

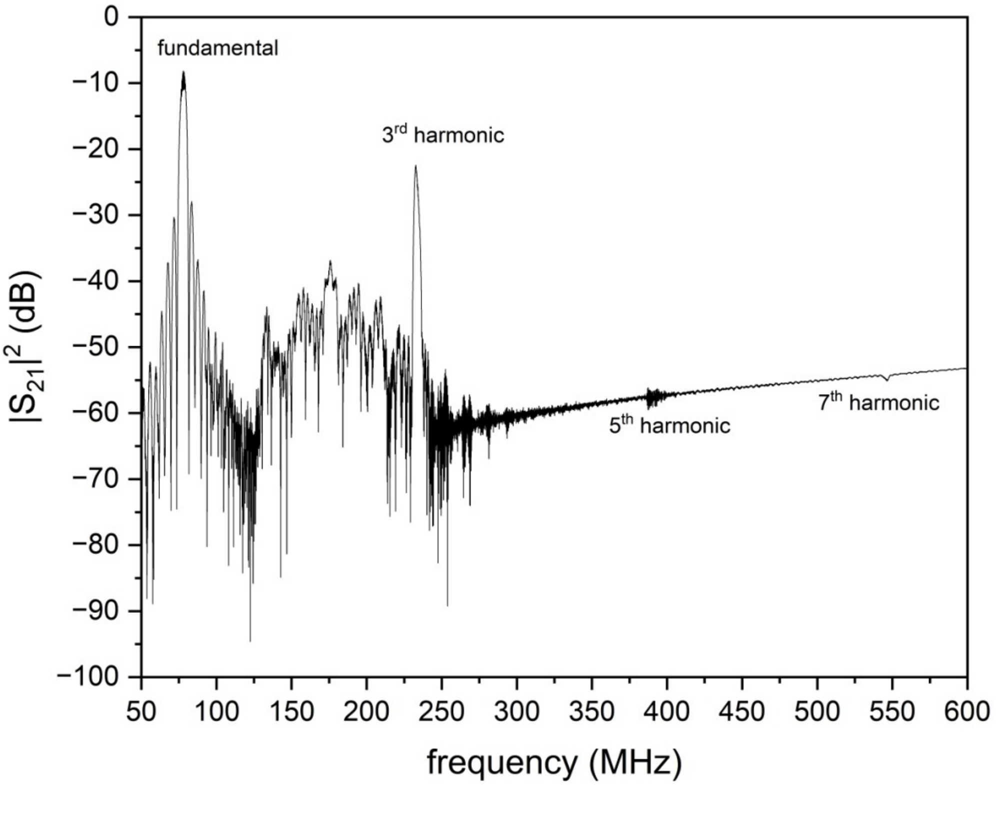

Figure S1. The measurement of the fundamental, 3rd, 5th and 7th harmonic amplitude for the sample with ~130 nm rrP3HT polymer film on $LiNbO_3$ Y-X substrate.

The details of the sensing materials preparation

Materials. 2,5-dibromo-3-hexylthiophene (TCI, ≥97%), *t*-butylmagnesium chloride (Sigma Aldrich, 2M ether solution), dichloro-[1,3-bis(diphenylphosphino)propane]nickel(II) (Ni(dppp)Cl_2) (Sigma Aldrich, ≥97%) were used as received, without further purification. Anhydrous tetrahydrofurane (ACROS Organics, 99.9%) was distilled over metallic sodium prior to use. All reactions were conducted under dry nitrogen or argon flow, in oven-dried glassware.

Regioregular PHT was synthesized via the McCullough GRIM method (doi: 10.1016/j.polymer.2005.05.035), modified as per the work of De Girolamo (doi: 10.1021/jp0741758): a dry 100 cm^3 three-necked flask, equipped with septum, a condenser, a gas capillary and a magnetic dipole, was purged with nitrogen and charged, via syringe, with 2,5-dibromo-3-hexylthiophene (6.13 mmol), anhydrous tetrahydrofurane (21.5 cm^3) and *t*-butylmagnesium chloride (7.46 mmol). The reaction mixture was refluxed for 2 h, followed by addition of the Ni(dppp)Cl_2 catalyst (0.0318 mmol) and heating for the next 1 h. The crude polymer was precipitated by quenching the reaction mixture in methanol.

Purification of crude products. Obtained polymer were purified with sequential Soxhlet extraction with methanol, hexane and chloroform. Vacuum-dried chloroform fractions were characterized by means of ^{1}H-NMR and SEC analyses and used as such in humidity sensing investigations.

Molecular characterisation of obtained products. ^{1}H-NMR analysis was performed from solutions in $CDCl_3$ on a Varian Unity Inova (Palo Alto, CA, USA) spectrometer with a resonance frequency of 300 MHz using TMS as internal standard. The number average molecular weights and dispersities were determined using a size-exclusion (SEC) chromatograph, equipped with an 1100 Agilent 1260 Infinity isocratic pump, an autosampler, a degasser, a thermostatic box for columns and a differential refractometric MDS RI Detector (Santa Clara, CA, USA). The molecular weight obtained by SEC was based on calibration with linear polystyrene standards (580-300,000 g/mol). Pre-column guard 5 μm (50 x 7.5 mm) and PLGel 5 μm MIXED-C (300 x 7.5 mm) column were used for separation. The measurements were carried out in THF (HPLC grade) as the solvent, at 30°C with a flow rate of 0.8 cm^3/min.

RR-PHT: ^{1}H-NMR ($CDCl_3$, 300 MHz) δH, ppm: 6.98 (s, 1H), 2.81 (m, 2H), 1.76-1.66 (m, 2H), 1.47-1.34 (m, 6H), 0.91 (t, J=6,9 Hz, 3H).

RR-PHT have been prepared, with molecular weights of 10,000 g/mol, and dispersities of 1.3 respectively (as determined by size exclusion chromatography, SEC).
